# BBX16, a B‐box protein, positively regulates light‐induced anthocyanin accumulation by activating *MYB10* in red pear

**DOI:** 10.1111/pbi.13114

**Published:** 2019-04-14

**Authors:** Songling Bai, Ruiyan Tao, Yinxin Tang, Lei Yin, Yunjing Ma, Junbei Ni, Xinhui Yan, Qinsong Yang, Zhongying Wu, Yanling Zeng, Yuanwen Teng

**Affiliations:** ^1^ Department of Horticulture Zhejiang University Hangzhou China; ^2^ Zhejiang Provincial Key Laboratory of Integrative Biology of Horticultural Plants Hangzhou China; ^3^ The Key Laboratory of Horticultural Plant Growth Development and Quality Improvement Ministry of Agriculture of China Hangzhou China; ^4^ Institute of Horticulture Henan Academy of Agriculture Sciences Zhengzhou China; ^5^ Key Laboratory of Cultivation and Protection for Non‐Wood Forest Trees Ministry of Education Central South University of Forestry and Technology Changsha China

**Keywords:** pear, anthocyanin accumulation, light, BBX16, MYB10

## Abstract

The red coloration of pear (*Pyrus pyrifolia*) results from anthocyanin accumulation in the fruit peel. Light is required for anthocyanin biosynthesis in pear. A pear homolog of *Arabidopsis thaliana BBX22*,* PpBBX16*, was differentially expressed after fruits were removed from bags and may be involved in anthocyanin biosynthesis. Here, the expression and function of *PpBBX16* were analysed. *PpBBX16*'s expression was highly induced by white‐light irradiation, as was anthocyanin accumulation. *PpBBX16*'s ectopic expression in *Arabidopsis* increased anthocyanin biosynthesis in the hypocotyls and tops of flower stalks. PpBBX16 was localized in the nucleus and showed trans‐activity in yeast cells. Although PpBBX16 could not directly bind to the promoter of *PpMYB10* or *PpCHS* in yeast one‐hybrid assays, the complex of PpBBX16/PpHY5 strongly trans‐activated anthocyanin pathway genes in tobacco. *PpBBX16*'s overexpression in pear calli enhanced the red coloration during light treatments. Additionally, *PpBBX16*'s transient overexpression in pear peel increased anthocyanin accumulation, while virus‐induced gene silencing of *PpBBX16* decreased anthocyanin accumulation. The expression patterns of pear *BBX* family members were analysed, and six additional *BBX* genes, which were differentially expressed during light‐induced anthocyanin biosynthesis, were identified. Thus, PpBBX16 is a positive regulator of light‐induced anthocyanin accumulation, but it could not directly induce the expression of the anthocyanin biosynthesis‐related genes by itself but needed PpHY5 to gain full function. Our work uncovered regulatory modes for *PpBBX16* and suggested the potential functions of other pear *BBX* genes in the regulation of anthocyanin accumulation, thereby providing target genes for further studies on anthocyanin biosynthesis.

## Introduction

Peel colour is an important aesthetic quality of pear (*Pyrus pyrifolia*) that directly affects consumer appeal (Zhang *et al*., 2013). Red‐coloured European pear has been widely cultivated and accepted worldwide, but green/yellow Asian pears still occupy the majority of the market (Sun *et al*., 2014). In recent years, along with the introduction of local red pear cultivars into breeding programs, red Asian pears have gradually increased in the market and have gained consumers’ acceptance (Zhang *et al*., 2013). However, unlike the European pear, which easily colours, the red Asian pear does not easily produce the red‐coloured peel, especially in the warmer regions of China (Bai *et al*., 2017).

The red coloration of Asian pear results from the accumulation of anthocyanin. Anthocyanin is biosynthesized through the flavonoid pathway, in which phenylalanine ammonia‐lyase (PAL), chalcone synthase (CHS), chalcone isomerase (CHI), flavanone‐3‐hydroxylase, dihydroflavonol 4‐reductase (DFR), anthocyanidin synthase (ANS) and UDP‐glucose: flavonoid 3‐glucosyltransferase (UFGT) are the key enzymes (Jin *et al*., 2016; Qian *et al*., 2014). The genes encoding these enzymes are transcriptionally regulated by a protein complex formed by an R2R3‐MYB, a basic‐helix‐loop‐helix and a WD repeat protein, namely the MBW complex (Xu *et al*., 2015). This complex binds to the promoters of structural genes and induces their expressions (Dubos *et al*., 2010). In rosaceous plants, a series of anthocyanin accumulation‐related MYB proteins have been identified, among which PpMYB10 (Feng *et al*., 2010) and PpMYB114 (Yao *et al*., 2017) control the red coloration of pear.

The transcriptional activation of structural genes by MYB proteins is the most important step in the regulation of anthocyanin biosynthesis. MYB proteins are transcriptionally and post‐translationally regulated by various internal and external factors (Allan and Espley, 2018). Specifically, the transcriptional alteration of *MYB* genes strongly correlates with anthocyanin accumulation (Xu *et al*., 2015). Among the various factors, light is indispensable for anthocyanin biosynthesis in pear (Sun *et al*., 2014). The mechanism of light‐induced anthocyanin biosynthesis has been well characterized in the model plant *Arabidopsis thaliana* as part of photomorphogenesis (Maier *et al*., 2013). It requires light‐responsive elements, including phytochromes and their downstream factors, such as CONSTITUTIVELY PHOTOMORPHOGENIC 1 (COP1) and LONG HYPOCOTYL 5 (HY5) (Lau and Deng, 2012). In pear, PpHY5 directly binds to the promoter of *PpMYB10*, but no solid evidence indicates transcriptional activity (Tao *et al*., 2018). However, the overexpression of apple *MdHY5* results in the overaccumulation of anthocyanin in apple calli (An *et al*., 2017). In addition, ERF, NAC and BBX proteins are involved in the transcriptional regulation of MYB proteins (Allan and Espley, 2018).

B‐box (BBX) proteins belong to the zinc finger protein super family and are characterized as containing at least one B‐box motif (Gangappa and Botto, 2014). In animals, the B‐box motif is always present with RING finger and coiled‐coil domains, and these proteins were named as TRIPARTITE MOTIF/RING, B‐box and coiled‐coil (Borden, 1998). These proteins are involved in transcriptional regulation, cell cycle regulation and ubiquitination (Laubinger *et al*., 2006). In plants, BBX proteins contain one or two B‐box domains near their N termini, and some proteins have a CONSTANS, CO‐LIKE and TOC1 motif (CCT domain) in their C termini (Crocco and Botto, 2013). The B‐box domains are important for BBX proteins, which affect protein‐protein interactions, nuclear bodies and transcriptional regulation (Qi *et al*., 2012). In *Arabidopsis*, 32 BBX proteins have been identified, and they are divided into five groups based on their sequences and structures (Gangappa and Botto, 2014). The BBX proteins have also been identified in other plant species, such as rice (*Oryza sativa*; Huang *et al*., 2012) and apple (*Malus × domestica*; Bai *et al*., 2014).

BBX proteins participate in numerous biological processes in plants, specifically in flower induction and photomorphogenesis. As part of photomorphogenesis, light‐induced anthocyanin accumulation involves several BBX proteins. AtBBX21 (Xu *et al*., 2016), AtBBX22 (Chang *et al*., 2008) and AtBBX23 (Zhang *et al*., 2017) are positive regulators of anthocyanin biosynthesis, while AtBBX24 (Job *et al*., 2018), AtBBX25 (Gangappa *et al*., 2013a) and AtBBX32 (Holtan *et al*., 2011) suppress anthocyanin accumulation. In most cases, BBXs directly transcriptionally regulate the anthocyanin biosynthesis‐related genes or indirectly regulate these genes through interactions with other proteins, such as HY5 (Gangappa and Botto, 2014). Furthermore, MdCOL11, the homolog of AtBBX22 in apple, may regulate anthocyanin biosynthesis, but solid evidence supporting the regulatory role of MdCOL11 in the anthocyanin biosynthesis is still lacking (Bai *et al*., 2014). In addition, BBX proteins also mediate hormonal signalling networks. AtBBX20 is involved in the brassinosteroid signalling pathway (Wei *et al*., 2016), and AtBBX18 promotes hypocotyl growth by increasing gibberellin levels (Wang *et al*., 2011). BBX21 can bind to the promoters of *ABA INSENSITIVE 5*, which is a hub of the abscisic acid signalling pathway, and regulate its expression (Xu *et al*., 2014).

After data mining our previously published transcriptome, a pear *BBX* gene, namely *PpBBX16*, was found to be differentially expressed in pear fruit after they were removed from bags, and *PpBBX16* may be involved in anthocyanin biosynthesis (Bai *et al*., 2017). In this work, the expression and function of the *PpBBX16* gene were analysed. By applying various approaches, we found that PpBBX16 indirectly activated the expression of *PpMYB10*, which subsequently activated anthocyanin biosynthesis. In addition, a genomewide analysis of the pear *BBX* gene family resulted in the further identification of other *BBX* genes that were differentially expressed during pear coloration. Our work resulted in a regulatory model for PpBBX16 and suggested the potential regulatory functions of other pear *BBX* proteins in the anthocyanin accumulation process, which helps us to understand the transcriptional regulation that occurs upstream of *PpMYB10* during anthocyanin biosynthesis.

## Results

### Identification and expression of *PpBBX16* under light conditions in ‘Red Zaosu’

In our previous transcriptome analysis, we identified the *PpBBX16* gene, which was the homolog of *A. thaliana BBX22* and was highly regulated by light exposure. Unexpectedly, when we cloned the *PpBBX16* gene, a highly similar gene, *PpBBX16‐2*, which was absent from the predicted pear transcripts, was also isolated from ‘Red Zaosu’ pear. The two proteins encoded by these genes shared a ~90% sequence identity (Figure S1a). A phylogenetic tree showed that *PpBBX16* and *PpBBX16‐2* were closely related to *MdBBX22*‐like genes (*MdBBX22‐1*,* MdBBX22‐2* and *MdBBX22‐3*) (Figure S1b).

**Figure 1:**
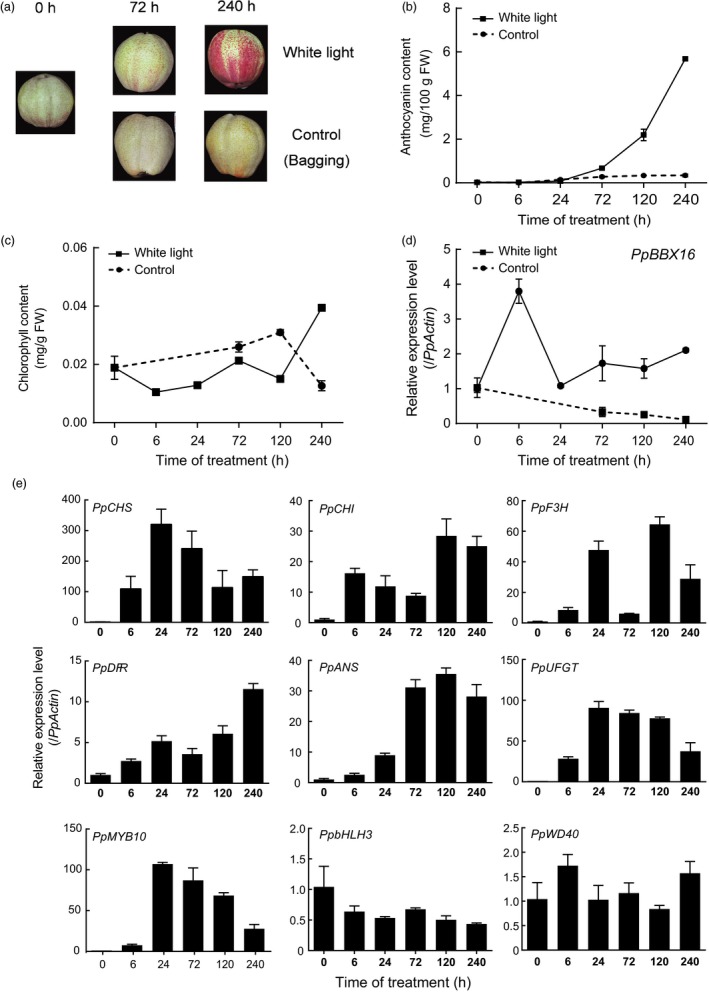
*PpBBX16*'s expression pattern during light treatment in ‘Red Zaosu’ pear. The bagging treatment was performed at 15 DAFB in the orchard. The light treatment was performed using the bagged fruit at 150 DAFB. (a) Colour changes of ‘Red Zaosu’ during the treatment. (b, c) Changes in anthocyanin (b) and chlorophyll (c) contents during the treatment. (d) *PpBBX16*'s expression pattern during the treatment. (e) Anthocyanin biosynthesis‐related genes’ expression profiles during the treatment. Error bars represent the standard deviations of three biological replicates.

The expression patterns of *PpBBX16* and the anthocyanin biosynthesis‐related genes during light exposure were further analysed. As shown in Figure 1, anthocyanin continuously accumulated after a 24‐h light treatment until the end of the experiment (240 h of light treatment), while a corresponding increase in chlorophyll was not recorded (Figure 1a–c). During the experiment, the expression of *PpBBX16* was immediately up‐regulated (within 6 h) after exposure to light (Figure 1d), while most of the anthocyanin biosynthetic structural genes were highly up‐regulated only after 24 h of treatment (Figure 1e), which was consistent with the anthocyanin accumulation. However, among the regulatory genes, only *PpMYB10* was up‐regulated by light exposure, while the *PpbHLH3* and *PpWD40* genes were not differentially expressed (Figure 1e).

### Ectopic expression of *PpBBX16* in *Arabidopsis*

**Figure 2:**
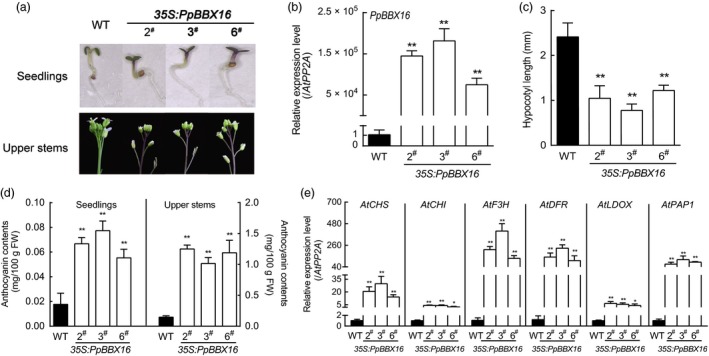
Effects of *PpBBX16*'s ectopic expression in *Arabidopsis*. (a) The phenotypes of wild‐type ‘Columbia 0’ and transgenic *Arabidopsis*. Three independent transgenic lines were used in the experiment. The anthocyanins overaccumulated in the seedlings (upper panel) and the tops of flower stalks (bottom panel) in the transgenic lines. (b) The *PpBBX16*'s expression level in transgenic *Arabidopsis* lines. (c) The ectopic expression of PpBBX16 resulted in shortened hypocotyls. (d) The anthocyanin contents in the transgenic lines. (e) The expression levels of anthocyanin biosynthesis‐related genes (*AtCHS*,* AtCHI*,* AtF3H*,* AtDFR*,* AtLDOX* and *AtPAP1*) in transgenic lines. The error bars represent the standard deviations of three biological replicates. Asterisks indicate significant differences (two‐tailed Student's *t*‐test, * *P* < 0.05, ** *P* < 0.01).

To verify the function of *PpBBX16,* a homolog of *AtBBX22*, it was ectopically expressed in wild‐type *Arabidopsis*. The transgenic lines showed stronger photomorphogenesis phenotypes, including shorter hypocotyls and greater anthocyanin accumulations (Figure 2a–d). However, the *PpBBX16*‐expressing transgenic lines also showed an overaccumulation of anthocyanins at the tops of the floral stems (Figure 2a,d). In the transgenic lines, the expression levels of anthocyanin biosynthesis‐related genes were highly regulated, which was consistent with the anthocyanin content (Figure 2e).

### Subcellular localization and trans‐acting activity of PpBBX16

**Figure 3:**
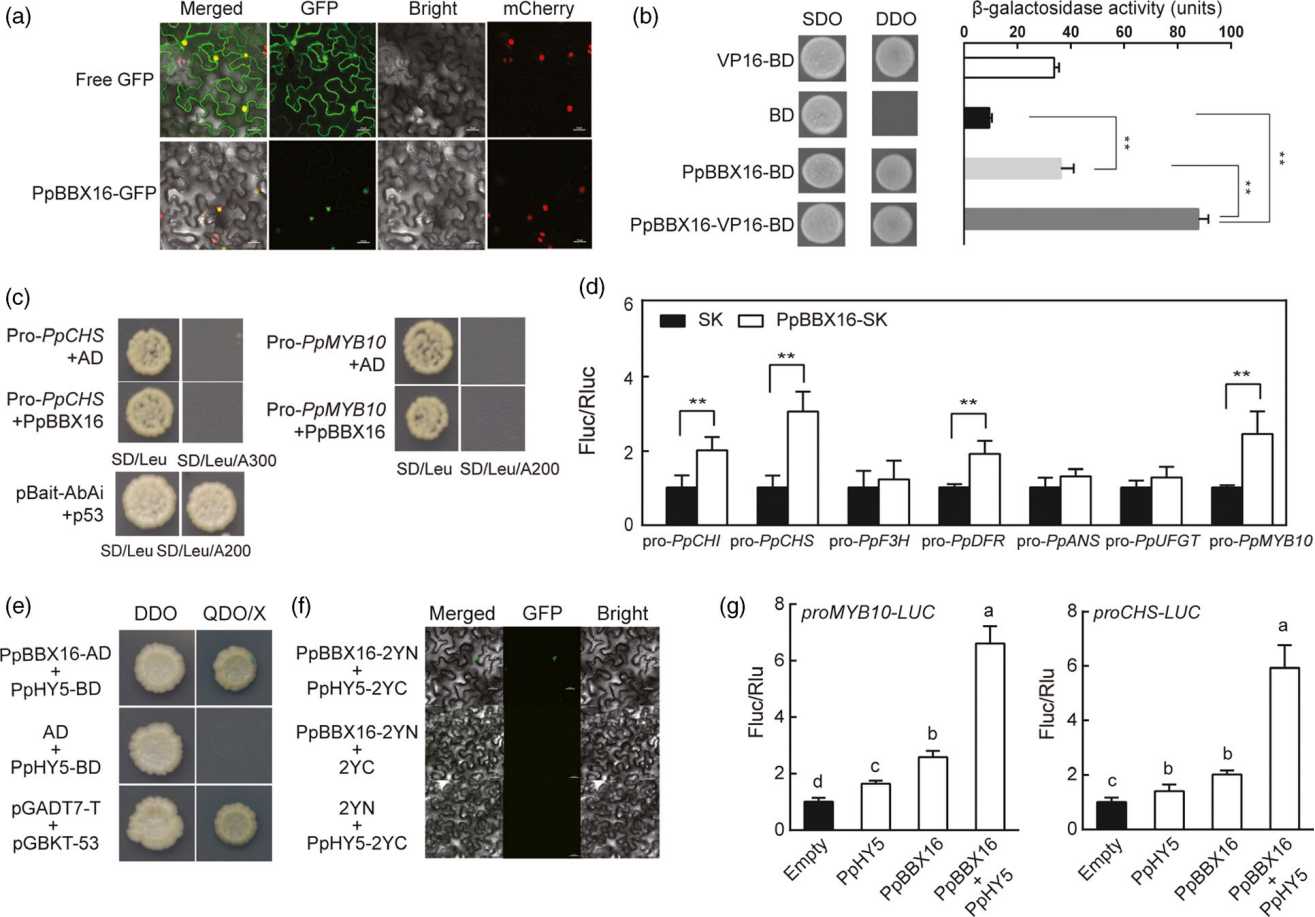
PpBBX16 and PpHY5 jointly activated the anthocyanin biosynthesis. (a) Subcellular localization of PpBBX16 expressed in tobacco leaf cells. (b) Trans‐acting activity of PpBBX16 transformed into yeast cells. The β‐galactosidase activities reflected the trans‐acting activities. (c) Yeast one‐hybrid assays of PpBBX16 and the promoter of *PpCHS* or *PpMYB10*. PpBBX16 could not interact with either promoter region, even though they harboured G‐boxes and can be directly bound by PpHY5 (Tao *et al*., 2018). (d) PpBBX16 transcriptionally induced the activity of anthocyanin biosynthesis‐related genes (*PpCHS*,* PpCHI*,* PpDFR* and *PpMYB10*) in dual‐luciferase assay. (e, f) PpBBX16 interacting with PpHY5. The physical interaction of PpBB16 and PpHY5 was tested by yeast two‐hybrid assays (e) and BiFC assays (f). (g) PpBBX16 and PpHY5 jointly promoted the expression of *PpMYB10* and *PpCHS*. Error bars for dual‐luciferase assays represent the standard deviation of three independent experiments each with six technical replicates. Lower case letters above bars indicate a significant difference determined by two‐way ANOVA followed by multiple comparisons with Tukey's test (*P* < 0.05). Asterisks indicate significant differences (two‐tailed Student's *t*‐test, * *P* < 0.05, ** *P* < 0.01).

When the PpBBX16‐GFP fusion protein was transiently expressed in the leaves of *N. benthamiana* with the nuclear expression of mcherry, orange fluorescent signals were observed in the nuclei. The GFP‐related fluorescence was distributed throughout the cell when the empty GFP protein was expressed (Figure 3a). Furthermore, PpBBX16‐BD showed a trans‐acting ability similar to that of VP16, and the activity was enhanced in BBX16‐VP16‐BD fusion proteins (Figure 3b). Thus, we confirmed that PpBBX16 is a transcription factor having an intact trans‐acting activity.

To determine how PpBBX16 induced anthocyanin biosynthesis, the correlations between PpBBX16 and anthocyanin biosynthesis‐related genes were analysed. The direct interactions between PpBBX16 and structural or regulatory genes could not be detected using the yeast one‐hybrid assay (Figure 3c). PpBBX16 could not bind to the promoter regions of either *PpCHS* or *PpMYB10*, although these regions contained G‐box motifs and can be directly bound by HY5, which binds to the same G‐box motif as BBX proteins. We further analysed the trans‐activation capability of PpBBX16 on the anthocyanin‐related genes in tobacco. When PpBBX16 and each of the *PpCHI*,* PpCHS*,* PpDFR* and *PpMYB10* promoter‐driven luciferase were co‐infiltrated, significantly greater luciferase activities were observed, which suggested that PpBBX16 was able to induce the expression levels of these genes (Figure 3d). As HY5 acted as a partner of BBX22 to promote photomorphogenesis in Arabidopsis, we further analysed the physical interaction between PpBBX16 and PpHY5. The results showed that PpBBX16 associated PpHY5 in yeast two‐hybrid assay and BiFC assay (Figure 3e,f) and such association enhanced the activation in the promoters of *PpMYB10* and *PpCHS* in dual‐luciferase assay (Figure 3g).

### Overexpression of *PpBBX16* in pear calli

**Figure 4:**
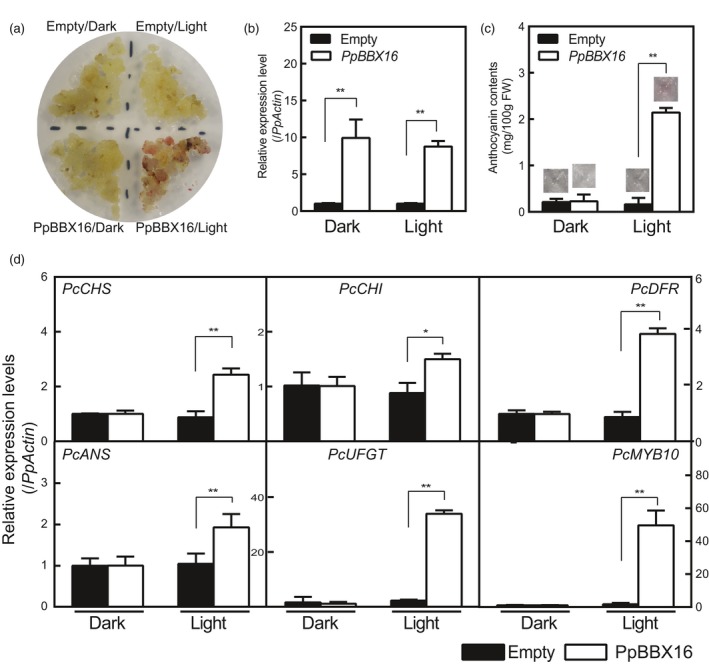
The effects of PpBBX16 overexpression in pear calli. (a) *PpBBX16*'s overexpression resulted in an anthocyanin accumulation after 2 days of the light treatment. (b) *PpBBX16*'s expression level in transgenic pear calli. (c) Anthocyanin contents in transgenic pear calli after the light treatment. The corresponding anthocyanin extracts are shown above each bar. (d) The expression levels of anthocyanin biosynthesis‐related genes (*PcCHS*,* PcCHI*,* PcDFR*,* PcANS*,* PcUFGT* and *PcMYB10*) in pear calli. The error bars represent the standard deviations of three biological replicates. Asterisks indicate significant differences (two‐tailed Student's *t*‐test, * *P* < 0.05, ** *P* < 0.01).

To further confirm the function of PpBBX16 in anthocyanin biosynthesis of pear, we overexpressed the *PpBBX16* gene in pear calli. The pear calli were induced from the flesh of European pear ‘Clapp's Favorite’ of fruitlets in our laboratory. Empty and *PpBBX16*‐containing calli are usually cultured in the dark and are white to light yellow in colour. When calli were moved to continuous white light, an overaccumulation of anthocyanin was observed after 2 days in *PpBBX16* calli but not in wild‐type calli (Figure 4a). As expected, the expression levels of *PpBBX16* were high under both dark and light conditions in transgenic calli, but the anthocyanin biosynthesis‐related genes were highly expressed only under light conditions (Figure 4b–d). These results indicated that PpBBX16 is involved in light‐induced anthocyanin biosynthesis.

**Figure 5:**
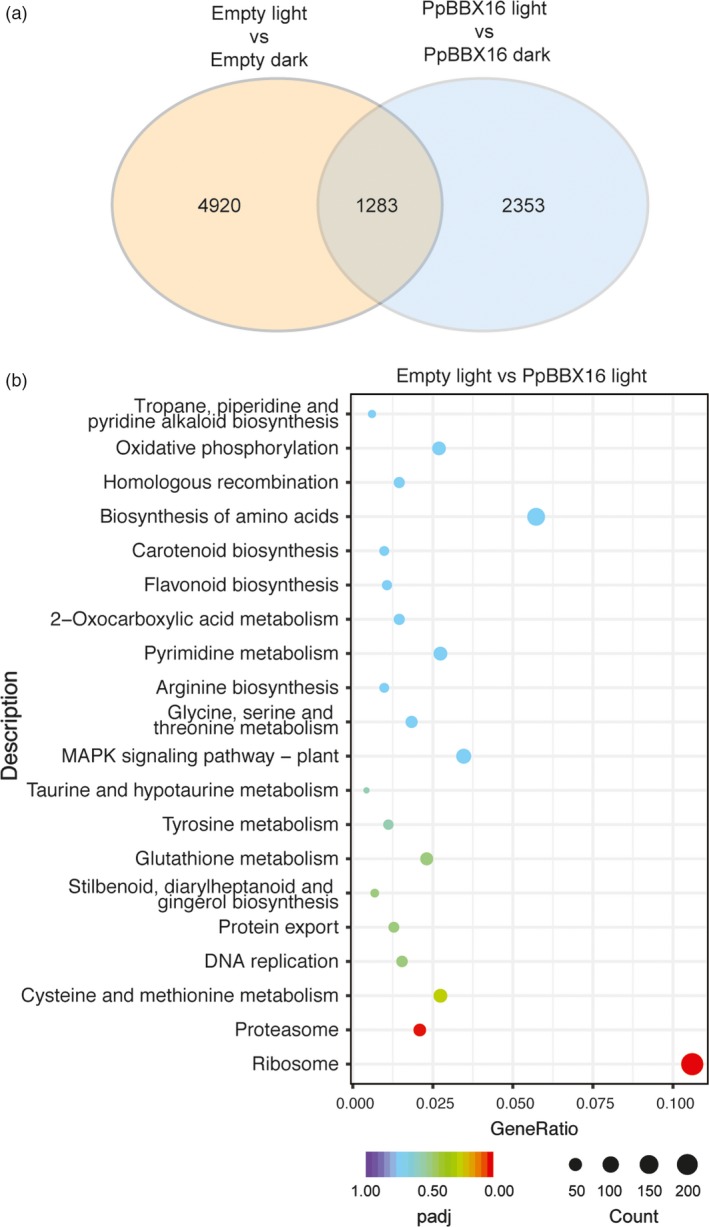
RNA‐Seq analysis of PpBBX16 overexpression calli under light/dark condition. (a) The Venn graph of the numbers of differential expression genes between Empty and PpBBX16 overexpression calli under dark/light conditions. (b) The enrichment analysis of KEGG pathways in the differential expression genes between Empty and PpBBX16 overexpression calli under light condition.

Furthermore, the Empty and *PpBBX16*‐overexpression calli were subjected to RNA‐Seq analysis (Figure 5). Light treatment induced the transcription changes of more than 6000 genes, among which 1283 genes overlapped with *PpBBX16*‐overexpression calli samples. On the other hand, 2353 genes were identified as light‐induced differentially expressed genes specifically in *PpBBX16*‐overexpression calli (Figure 5a). Furthermore, the KEGG enrichment analysis was carried out in the differential expression genes between Empty and *PpBBX16*‐overexpressive calli under light treatment. The results showed that besides flavonoid biosynthesis pathway, other biological pathways, such as carotenoid biosynthesis, biosynthesis of amino acids, *etc*., also showed relatively high *P*‐value in this analysis (Figure 5b). These results further confirmed that PpBBX16 was involved in the flavonoid biosynthesis and other pathways as well.

### Transient expression of *PpBBX16* in pear fruit

**Figure 6:**
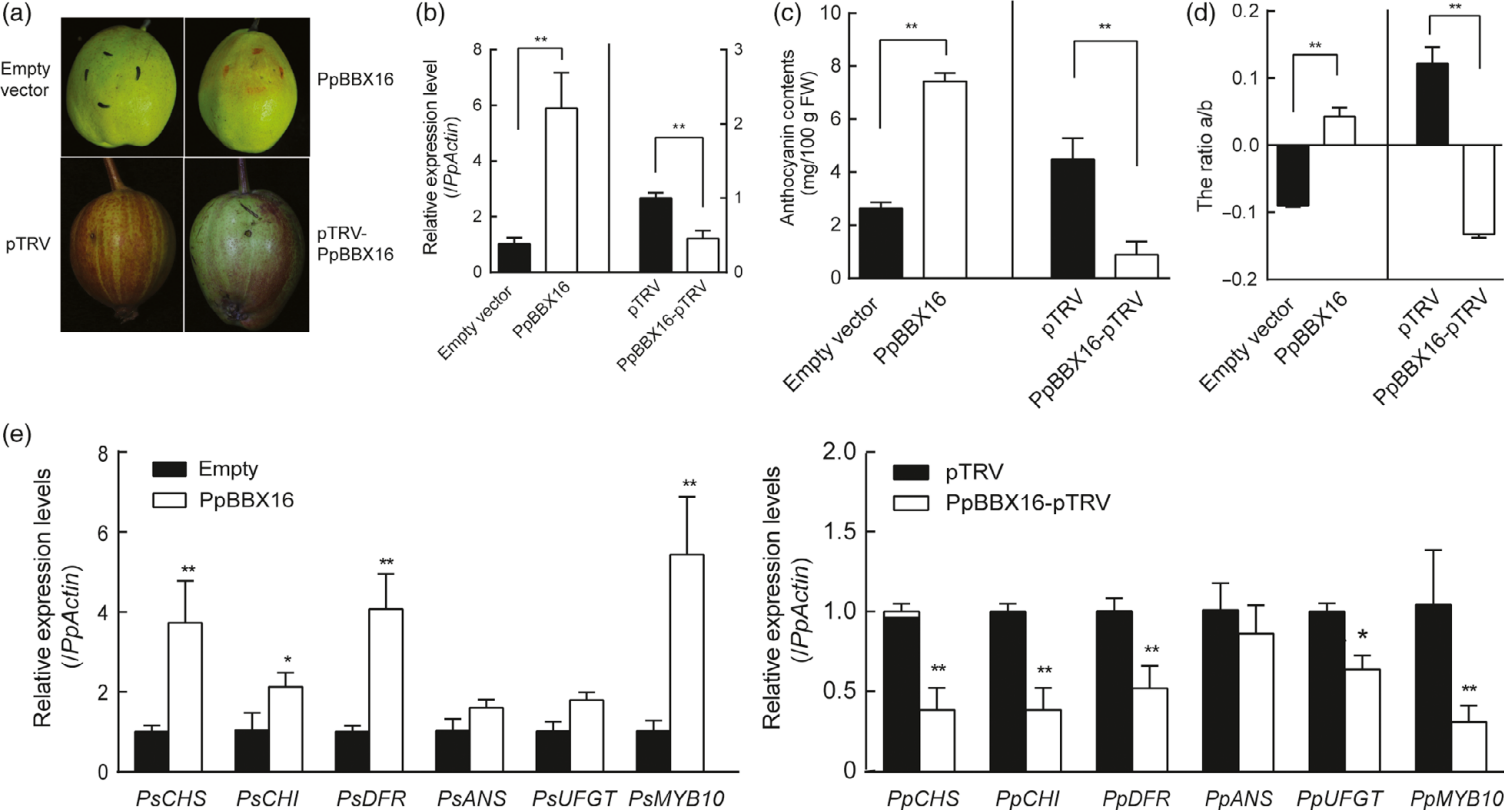
Effects of the transient expression or silencing of *PpBBX16* in pear fruit. (a) The transient expression of *PpBBX16* in the fruit of ‘Korla’ pear induced anthocyanin biosynthesis, while the silencing of *PpBBX16* suppressed anthocyanins’ accumulation. (b–d) The expression patterns of *PpBBX16* (b), the anthocyanin contents (c) and the colour changes (d) of the fruit peels were analysed. (e) The expression levels of anthocyanin biosynthesis‐related genes were also affected by the transient expression and the silencing of *PpBBX16* in fruit peels. The error bars represent the standard deviations of three biological replicates. Asterisks indicate significant differences (two‐tailed Student's *t*‐test, * *P* < 0.05, ** *P* < 0.01).

Because the calli were induced from the flesh of European pear, to further confirm the involvement of PpBBX16 in anthocyanin biosynthesis in the pear peel, we transiently overexpressed *PpBBX16* in ‘Korla’ pear and silenced it in ‘Red Zaosu’ pear. The transient overexpression of *PpBBX16* in ‘Korla’ pear induced anthocyanin biosynthesis surrounding the injection site after 5 days of light treatment, while no red colour was observed after the injection of *Agrobacteria* containing the empty vector. In contrast, after silencing *PpBBX16* in ‘Red Zaosu’ fruitlets, anthocyanin biosynthesis was suppressed around the injection site (Figure 6a–d). A further analysis showed that the expression of *PpBBX16* and anthocyanin biosynthetic‐related genes were in accordance with the anthocyanin accumulation (Figure 6e).

Because many members of the *BBX* family have been characterized as light‐responsive genes in model plants, we further analysed the *BBX* family in pear and monitored their expression levels during light‐induced anthocyanin biosynthesis. In addition to the 37 BBX proteins identified from published pear genome data, we isolated two additional BBX proteins, PpBBX16‐2 and PpBBX21‐2, by homologous cloning. In total, 39 BBX family members were identified in pear and were named based on their chromosomal distribution (Figure S2). On the basis of the numbers and distributions of B‐box and CCT domains, these BBX proteins were divided into five groups (Figures S2 and S3). Group I contained 11 members that contained two B‐box domains in their N termini and one CCT domain near their C termini. Groups II and IV had 11 and 6 members, respectively, with a reverse B‐box domain. Groups III (four members) and V (six members) were identified as lacking the CCT domain (Figure S3). qPCR was performed to determine the expression patterns of *BBX* gene family members during light exposure at 0, 6, 24, 72, 120 and 240 h. *PpBBX1*,* PpBBX8*,* PpBBX14, PpBBX16‐2*,* PpBBX35* and *PpBBX36* showed sharp increases in expression during the first 24 h, and *PpBBX9* and *PpBBX17* reached their maximum expression levels by 6 h of light treatment. On the contrary, several genes had greater expression levels during the latter stage of the treatment. The expression levels of *PpBBX2*,* PpBBX4*,* PpBBX10*,* PpBBX22*,* PpBBX23*,* PpBBX24*,* PpBBX26*,* PpBBX28* and *PpBBX32* peaked at 120 h. However, the expressions of some *BBX* genes, such as *PpBBX5*, showed little change during the entire experiment (Figure S4). The primers are listed in Table S1.

## Discussion

Multiple lines of evidences showed that although apple and pear are genetically related, their regulation of anthocyanin biosynthesis seems different. Firstly, UV‐B irradiation is less effective on pear than that on apple (Zhu *et al*., 2018). Secondly, apple and pear showed opposing effects of some hormones, such as jasmonic acid and ethylene. Therefore, we speculated that the regulation pathway of pear anthocyanin biosynthesis might be different from that in apple. To uncover such differences, we have clarified the protein‐protein interactions and protein‐DNA interactions among PpCRYs, PpCOP1, PpHY5 and PpMYB10 within the conserved light‐responsive pathway in red pear (Tao *et al*., 2018), but could not find any differences. In the present work, we further analysed light‐responsive B‐Box proteins in pear.

### PpBBX16 responds to light and induces anthocyanin biosynthesis

Light is required for pear fruit coloration (Sun *et al*., 2014). In our previous reports, we confirmed the presence of the conserved light‐induced pathway, including the COP1‐HY5‐MYB10 regulatory module that is responsible for anthocyanin biosynthesis, in the red pear cultivar ‘Red Zaosu’, but this model could still not fully explain its red coloration. The sequences of these genes and their promoter regions did not show obvious differences between ‘Red Zaosu’ and its original green cultivar ‘Zaosu’, suggesting that other regulatory factors were involved (Tao *et al*., 2018). In the present work, a transcription factor belonging to the BBX family, *PpBBX16*, was found to induce anthocyanin biosynthesis in heterogeneous and homogeneous systems by activating anthocyanin structural genes, suggesting that PpBBX16 contributes to the red coloration of pear. PpBBX16 is a homolog of *Arabidopsis* BBX22. AtBBX22 positively affects photomorphogenesis under the regulation of HY5 and COP1 on the transcriptional and post‐translational levels (Datta *et al*., 2008). We analysed PpBBX16's function and found that it efficiently enhanced light‐induced anthocyanin accumulation (Figures 2, 4, 5 and 6). The overexpression of PpBBX16 induced anthocyanin overaccumulation in the hypocotyls of transgenic *Arabidopsis*, indicating that PpBBX16's role in inducing anthocyanin biosynthesis was intact (Figure 2). Similarly, apple COL11 (a homolog of *Arabidopsis* BBX22) showed a similar role in the regulation of anthocyanin accumulation (Bai *et al*., 2014). PpBBX16 is a nuclear‐localized protein (Figure 3a), which is important for its function as a transcription factor. Indeed, PpBBX16 showed a strong trans‐activation activity in yeast, which indicated that PpBBX16 positively regulated downstream genes (Figure 3b). Thus, PpBBX16 activated the expression of several structural and regulatory genes, such as *PpCHI*,* PpCHS*,* PpDFR* and *PpMYB10* (Figure 3e), which is consistent with some reports that *BBX* genes in *Arabidopsis* can increase anthocyanin biosynthesis by regulating anthocyanin biosynthesis‐related genes’ expression levels (Datta *et al*., 2008). In transgenic *Arabidopsis*, these anthocyanin biosynthesis genes were also activated by exogenous *PpBBX16* (Figure 2e). To confirm that *PpBBX16* contributed to anthocyanin biosynthesis, it was overexpressed in pear flesh‐originated calli, which showed that a high level of *PpBBX16* expression mediated the light‐induced red coloration of calli under light conditions (Figure 4). RNA‐Seq analysis using the calli indicated the enrichment of flavonoid pathway in differential expression genes between light treated Empty and PpBBX16 calli (Figure 5). Further transient assays involving pear peel also confirmed that *PpBBX16* was a positive regulator of anthocyanin biosynthesis (Figure 6). These results, together with the results from heterogeneous system, indicated that *PpBBX16* is a crucial player in the process of light‐induced anthocyanin biosynthesis in pear.

### PpBBX16 interacts with PpHY5 and induces the expression of anthocyanin structural genes and transcription factors in light

PpBBX16 could not directly bind the promoter of *PpCHS* or *PpMYB10* in the yeast one‐hybrid assay (Figure 3e) even such fragments used in this assay contained G‐box motifs but can be bound by PpHY5 (Tao *et al*., 2018). BBX family members in Arabidopsis, such as AtBBX21, have been identified to bind to the G‐box motif and are involved in the photomorphogenesis (Xu *et al*., 2016), while the apple BBX22 homolog, MdCOL11, potentially binds an unknown motif because a deletion assay identified a region without a G‐box that corresponds to the trans‐acting activity of MdCOL11 (Bai *et al*., 2014). The present work, however, failed to observe the physical interaction between PpBBX16 and *PpMYB10* promoter, but PpBBX16 was able to induce the promoter activities of *PpCHS*,* PpMYB10* and other anthocyanin‐related genes in the dual‐luciferase assay. The further analysis showed that PpBBX16 and PpHY5 physically interacted with each other *in vivo* and *in vitro* and the PpBBX16/PpHY5 complex strongly induced the promoter activity of *PpMYB10* (Figure 3e–g). Therefore, we proposed that full function of PpBBX16 might require the assistance of other transcription factor(s), such as HY5 (Figure 3e–g and Datta *et al*., 2008) to provide the DNA binding activity. However, PpBBX16 itself had the trans‐acting activity, at lease in yeast (Figure 3b), suggesting it might function by itself in a PpHY5‐independent manner. RNA‐Seq analysis identified many other KEGG pathways modulated by the overexpression of PpBBX16 (Figure 5), suggesting that PpBBX16 also functioned in other biological pathways.

### Potential functions of other *BBX* family members in anthocyanin accumulation

*BBX* genes have been identified in *Arabidopsis* (Crocco and Botto, 2013), rice (*O. sativa* L. ssp*. Japonica*) (Huang *et al*., 2012) and other plants (Almada *et al*., 2009). In this work, we identified 39 *BBX* family members in pear (Figures S2 and S3), which was more than in Arabidopsis (32) or rice (30) (Wu *et al*., 2012). However, because the pear genome is 3.9 times larger than that of *Arabidopsis*, the number of *BBX* family members is not consistent with the greater genome size, indicating that the *BBX* family expanded to various degrees among different species, probably owing to the different genome duplication events. Additionally, several BBX proteins were not correctly predicted in the published pear genome, such as PpBBX16‐2 and PpBBX21‐2, which suggested that the pear BBX family might have more members. Because tandem and large‐scale segmental duplications affect gene family expansion during plant evolution (Cannon *et al*., 2004), to detect the origins of duplicated BBX family genes in pear, we analysed the collinearity of the pear BBX family. However, no collinearity was found within the pear BBX family, probably as a result of the limited number of BBX members in the pear genome (Figure S5).

Several BBX family members regulate photomorphogenesis, including anthocyanin accumulation. In *Arabidopsis*,* AtBBX4*,* AtBBX20*,* AtBBX21* and *AtBBX22* have contradictory functions (Gangappa and Botto, 2014). The B‐box domains play crucial roles in the interactions with other proteins. In *Arabidopsis*,* AtBBX21* (Xu *et al*., 2016), *AtBBX22* (Datta *et al*., 2008), *AtBBX24* (Job *et al*., 2018) and *AtBBX25* (Gangappa *et al*., 2013a) physically interact with HY5, and three BBX proteins interact with HOMOLOG OF HY5 through B‐box domains (Gangappa *et al*., 2013b). A point mutation in the B‐box domain impedes the interaction with HY5 (Datta *et al*., 2007). Additionally, BBX proteins interact with other BBX family members. AtBBX32 interacts with AtBBX21 and suppresses its binding to HY5 (Holtan *et al*., 2011). Therefore, pear BBX proteins may interact with HY5 or other BBX proteins to induce anthocyanin biosynthesis.

Several BBX family members are regulated by light. The expression levels of *PpBBX1*,* PpBBX8* and *PpBBX35* peaked 24 h after the light treatment and then decreased (Figure S4), which was the same as some anthocyanin biosynthesis‐related genes, such as *PpPAL*,* PpUFGT*,* PpCHS* and *PpMYB10* (Figure 1e). However, *PpBBX4* and *PpBBX26* peaked 120 h after the light treatment (Figure S4). The expression of *PpBBX16* increased at 6 h after the light treatment (Figure 1d), which was prior to the increased expression of anthocyanin biosynthesis‐related genes and might control those genes’ expression. In addition, the sequence of *PpBBX16‐2* was very similar to that of *PpBBX16*. However, they had totally different expression patterns, with *PpBBX16* peaking at 6 h, while *PpBBX16‐2* peaked at 72 h. This might be attributed to the promoter differences that resulted in different expression patterns (Figure S4). PpBBX16‐2 was not found in the genome data, even though it highly similar to MdBBX22, which indicated that BBX family might be larger than predicted by the genome data (Figure S1).

### The usage of pear calli in the gene function studies of pear

Since apple calli were firstly used in the studies of anthocyanin‐related genes (Xie *et al*., 2012), they have been widely adopted in gene function studies in the apple, including flavonoid biosynthesis (Hu *et al*., 2016), the fruit acidity (Zhao *et al*., 2016), fruit ripening (Li *et al*., 2017) and abiotic stress (Sun *et al*., 2018). The use of apple calli enabled the researchers to easily observe effect of genes in the homologous system, thus greatly accelerating the study of gene functions in apples. Recently, the application of citrus calli has also been reported (Lu *et al*., 2018). The present work firstly reported the application of pear calli for the study of gene function and PpBBX16 was verified to successfully increase the anthocyanin biosynthesis in the transgenic pear calli (Figure 4). We believe that pear calli reported herein will advance the study of gene function in *Pyrus* species.

## Conclusions

**Figure 7:**
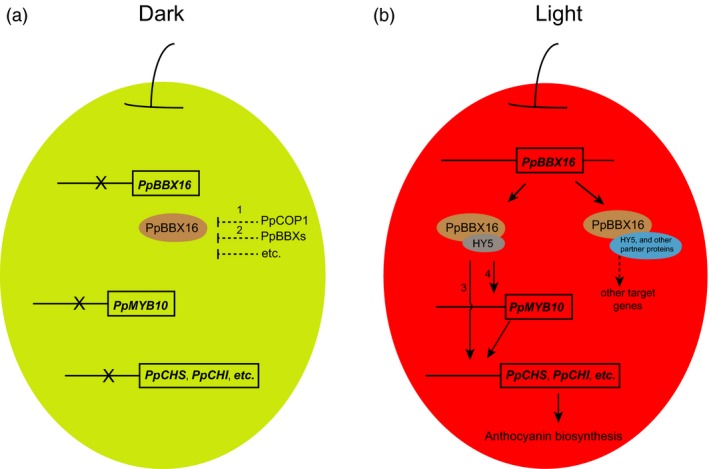
Proposed model of the mechanism of regulating anthocyanin accumulation through PpBBX16 in pear. (a) Red pear under dark conditions cannot accumulate anthocyanin. The dashed lines represent pathways already reported in *Arabidopsis*: Line 1, BBX proteins could be degraded through the 26S proteasome pathway by interactions with E3 ubiquitin ligases, such as COP1 (Datta *et al*., 2008). Line 2, The functions of BBX members can be suppressed by other BBX proteins (Holtan *et al*., 2011). (b) Red pear under light conditions accumulate anthocyanin through the *PpBBX16*‐involved pathway: PpBBX16 and PpHY5 jointly activate the expression of structural genes (Arrow 3) and *PpMYB10* (Arrow 4), which further induces the expression levels of structural genes.

Here, we identified a BBX family member, PpBBX16, which, along with the well‐characterized COP1–HY5–MYB10 regulatory module, is involved in light‐induced anthocyanin biosynthesis. Moreover, other BBX proteins were shown to be involved in light responses and anthocyanin accumulation (Figure 7). Our results provide a series of new target genes involved in the determinant of red coloration in pear that are located upstream of *PpMYB10*.

## Experimental procedures

### Plant materials and light treatment

Six‐year‐old trees of red pear cultivar ‘Red Zaosu’ in the orchard of the Institute of Horticulture, Henan Academy of Agricultural Sciences (E 113.71°, N 34.71°), were used as materials. The fruitlets were covered with double‐layered light‐proof paper bags 15 days after full bloom (DAFB), and mature fruits were harvested with bags at 150 DAFB. Approximately 300 uniform, defect‐free fruits were collected for postharvest light treatments. Half of these fruits were bag‐removed while the other half remained with the bag. All of these fruits were then treated in the phytotron at 17 °C with continuously white light (699 lux). The fruit peels were collected after 0, 6, 24, 72, 144 and 240 h of light treatment, immediately frozen in liquid nitrogen and stored at ‐80 °C until use.

### Colour and pigment measurements

Measuring the fruits’ colour was carried out with a chroma meter (CR‐400, Konica Minolta, Tokyo, Japan) at four evenly distributed equatorial sites.

The anthocyanins of fruit and pear calli were extracted according to Bai’ s protocol with minor modifications (Bai *et al*., 2014). In brief, 0.1 g of frozen peels was placed in 1 mL methanol: acetic acid (99 : 1, v : v) overnight in the dark at 4 °C. The absorbance of each 100‐μL sample extracted was assessed using a spectrophotometer (DU800, Beckman Coulter, Brea, CA, USA) at 530, 620 and 650 nm. The anthocyanin content was calculated using the formula: Absorbance = [(A_530_‐A_650_)‐0.2 × (A_650_‐A_620_)]/0.2.

To measure the anthocyanin pigments in *Arabidopsis*, 15 plants were frozen in liquid nitrogen and ground. The powders were immersed in 1 mL methanol: acetic acid (99 : 1, V : V) overnight in the dark at 4 °C. After centrifugation (13 400 ***g***), the liquid phase was used to test the absorption at 530 nm using a spectrophotometer.

Total chlorophyll was extracted and measured using the method described by Huang *et al*. (2009). A total of 0.5 g fruit peel was homogenized in 3 mL 80% cold acetone. Then, the mixture was centrifuged at 4 °C and 9400 ***g*** for 20 min. The absorbance of the extract was measured using a DU800 spectrophotometer at 645 and 663 nm. The total chlorophyll content was calculated using the formula: chlorophyll_total_ = (20.2 × A_645_) + (8.02 × A_663_).

### Pear fruit RNA isolation and reverse transcription quantitative PCR (qPCR)

Total RNA of fruit peel was extracted using a modified CTAB method according to Zhang *et al*.'s protocol (2013). First‐strand cDNA was synthesized from 4 μg DNA‐free RNA using the oligo (dT) PrimeScript™ RT reagent Kit with gDNA Erase (RR047Q, TaKaRa, Otsu, Japan) following the manufacturer's instructions. The cDNA was diluted threefold and used as the template for gene cloning and qPCR analysis. qPCR was performed using a CFX96 real‐time PCR detection system (CFX96, Bio‐Rad, Hercules, CA, USA). A template‐free negative control for each primer pair was included for each run. All relative expression levels were calculated using the $2^{-\Delta \Delta C_{t}}$ method against the pear *Actin* or Arabidopsis *PP2A* (JN684184 or AT1G13320, respectively) gene. The analysis was performed with three biological replicates. The primers were designed using the Primer‐BLAST tool (http://www.ncbi.nlm.nih.gov/tools/primer-blast/). The amplification efficiencies and product specificities of the primers were determined using standard curves and PCR‐product sequencing. The primers used for qPCR are listed in Table S1.

### Trans‐acting activity assay

For the trans‐activation activity assay, the complete CDS of *PpBBX16* was fused with the VP16 fragment (PpBBX16:VP16). PpBBX16 and PpBB16:VP16 were then independently ligated into the pGBKT7 (BD) vector. The constructed vectors, together with the positive (VP16‐BD) and negative (BD) controls, were independently transformed into yeast strain AH109 using the Yeastmaker™ Yeast Transformation System 2 (TaKaRa). The β‐galactosidase activity was detected based on He *et al*.'s protocol (2012) with minor modifications. The activity was analysed in three independent experiments with at least three biological replications for each assay.

### Subcellular localization analysis

The subcellular localization of PpBBX16 was determined using the method of Yang *et al*. (2018). In brief, the full‐length CDSs (without termination codons) of the target genes were independently cloned into the pCambia1300 vector, which contained the CaMV 35S promoter and *GFP* gene, resulting in fusion genes driven by the 35S promoter (primer listed in Table S1). *Agrobacteria tumefaciens* lines harbouring the vectors were independently infiltrated into the leaves of *Nicotiana benthamiana* transgenic lines containing red fluorescent protein expression in the nuclei. The fluorescence was observed by confocal laser scanning microscopy (A1, Nikon, Tokyo, Japan).

### Dual‐luciferase assay

Dual‐luciferase assays were performed with tobacco (*N. benthamiana*) in accordance with a previous publication (Niu *et al*., 2016). In brief, the full‐length CDS of the *BBX* gene was cloned into pGreenII 0029 62‐SK, while the promoter sequence of the *cis* gene was inserted into pGreenII 0800‐LUC. Both constructs were individually transformed into *A. tumefaciens* GV3101 (containing the pSoup vector) using the freeze–thaw method. *Agrobacterium* infiltration was carried out with a needle‐free syringe. The Firefly luciferase and Renilla luciferase activities were analysed 72 h after infiltration using the Dual‐Luciferase Reporter Assay System (E710, Promega, Madison, WI, USA) with Modulus Luminometers (GloMax96, Promega). Both luciferase activities were analysed in three independent experiments with at least six biological replications for each assay.

### Yeast one‐hybrid assays

Yeast one‐hybrid assays were performed using the Matchmaker Gold Yeast One‐Hybrid System Kit (TaKaRa) according to the manufacturer's protocol. Briefly, promoter fragments were ligated into the pAbAi vector, and PpBBX16 was cloned into the pGADT7 vector. The pAbAi vector was linearized and transformed into the yeast strain Y1HGold. Transformants were selected on plates containing a selective synthetic dextrose medium lacking uracil. The prey vectors were then transformed into Y1HGold cells harbouring the pAbAi‐bait and tested on SD/‐Leu/AbA plates.

### Yeast two‐hybrid assays

The yeast two‐hybrid assays were performed using the Matchmaker^®^ Gold Yeast Two‐Hybrid System Kit (TaKaRa, Dalian, China) in accordance with the manufacturer's instructions with a minor modification. Briefly, 500 ng pGADT7 (AD) and 500 ng pGBKT7 (BD) vectors were simultaneously transformed into Y2HGold yeast cells using the polyethylene glycol/lithium acetate method. The transformants were selected on QDO/X plates (SD/‐Trp/‐Leu/‐His/‐Ade/X‐α‐gal). The pGADT7‐T and pGBKT7‐53 vectors provided with the kit were used as positive controls.

### Bimolecular fluorescence complementation (BiFC) assays

For bimolecular fluorescence complementation (BiFC) assays, the coding sequences (CDSs, without the termination codon) were cloned into the YFP^N^ (p2YN) or YFP^C^ (p2YC). Then, *A. tumefaciens* strain GV3101 harbouring the constructs was transiently co‐expressed in all possible combinations of p2YN and p2YC fusion proteins in *Nicotiana benthamiana* leaves. Fluorescence was observed according to subcellular localization assays.

### Transient transformation analysis in pear fruit

For the transient overexpression assay, the mature fruit of ‘Korla’ pear (*Pyrus sinkiangensis,* the hybrid species of *Pyrus communis* and East Asian pear cultivars) was infected. ‘Korla’ pear trees bear fruit that are blushed on the side exposed to sun, which indicates the presence of an intact anthocyanin biosynthesis pathway in this cultivar, which makes it a suitable material for observing the induction of coloration. The fruits were infiltrated with the pCambia1301‐PpBBX16 vector in *A. tumefaciens* ‘GV3101’ with injection syringes according to Li *et al*.'s protocol with slight modifications (Li *et al*., 2012). Briefly, *A. tumefaciens* was grown to saturation in Luria–Bertani medium. After centrifugation (13 400 ***g***), the pellet was resuspended in the infection solution (10 mM MgCl_2_, 10 mM MES and 150 mM acetosyringone), and the mixture was kept at room temperature for 1 h. After infiltration, the fruits were kept under dark conditions for 1 day and then treated with continuous white light for 5 days. The empty pCambia1301 vector was used as the negative control (Empty). After images were taken, the fruit peel surrounding the injection sites was collected, immediately frozen in liquid nitrogen and stored at −80 °C until use.

For the virus‐induced gene silencing (VIGS) assay, a *PpBBX16* fragment (417‐772 bp) was amplified using the primer pair BBX16VIGS‐F/BBX16VIGS‐R and cloned into the pTRV2 vector (pTRV2‐PpBBX16). The resulting vector was introduced into *A. tumefaciens* strain EHA105 and then used for pear fruit infiltration. The ‘Red Zaosu’ pear fruitlets (50 DAFB) were used for the VIGS assays because they can achieve a full dark red colour, making them ideal for the analysis of coloration inhibition. The injection solution harbouring pTRV2‐PpBBX16 was co‐infiltrated with pTRV1 in fruitlets of ‘Red Zaosu’ pear on the tree. Then, 10 days after infiltration, the peels were collected, immediately frozen in liquid nitrogen and stored at −80 °C until use. Empty pTRV1 and pTRV2 were co‐infiltrated as the negative control (pTRV).

### Induction of pear calli and their transformation

The pear calli were induced from the flesh of young ‘Clapp's Favorite’ (*P. communis*) fruit on the NN69 (NITSCH and NITSCH 1969) solid medium with addition of sucrose (30 g/L), 6‐benzylaminopurine (0.5 mg/L) and 2,4‐dichlorophenoxyacetic acid (1.0 mg/L). The first‐generation calli were subcultured several times, and the rapidly growing soft calli were screened and maintained under dark condition on the MS (Murashige and Skoog) solid medium supplemented with sucrose (30 g/L), 6‐benzylaminopurine (0.5 mg/L) and 2,4‐dichlorophenoxyacetic acid (1.0 mg/L). The transformation of pear calli was performed as follows: pear calli were soaked in *A. tumefaciens* strain EHA105 (0.5 OD_600_) containing either the pCambia1301‐PpBBX16 vector or the empty pCambia1301 vector for 10 min. After 3 days coculture, the calli were then screened on MS solid medium mentioned above by 10 mg/L hygromycin B under continuous dark conditions at 24 °C. For the light treatment, the newly subcultured empty or *PpBBX16*‐containing calli were moved to the light conditions (16‐h light/8‐h dark) for 2 day and then used for observation.

### RNA‐Seq analysis

We used the pear calli harbouring Empty vector and pCambia1301‐PpBBX16 vector for RNA‐Seq analysis. The calli were incubated under dark or light for 2 days for RNA‐Seq. The calli of three different lines were simultaneously treated and used as biological replicates. The total RNAs were extracted as described above. Ten micrograms of total RNA each was used for next‐generation sequencing. The library construction and sequencing were performed by Novogene (Beijing, China) using the HiSeq X (Illumina, San Diego, CA) platform with a 150‐bp pair‐end strategy. The clean reads were mapped to the European pear genome sequence (Chagné *et al*., 2014; http://www.rosaceae.org) using HISAT2 (Pertea *et al*., 2016) with default parameters. The reads were then assembled into transcripts and compared with reference gene models using StringTie (Pertea *et al*., 2016). The differentially expressed gene analysis was performed using DESeq2 (Love *et al*., 2014).

### *Arabidopsis*’ hypocotyl measurement

Seeds of wild‐type (Col‐0) *A. thaliana* and overexpression lines (35S:*PpBBX16*) in the Col‐0 background were grown on 1/2 MS medium. Seeds were subjected to a chilling treatment at 4 °C for 72 h and then transferred into white light at 24 °C under long‐day conditions (16‐h light/8‐h dark). Five‐day‐old *Arabidopsis* seedlings were used for hypocotyl measurements. At least 10 seedlings were imaged, and hypocotyl lengths were measured using ImageJ software (https://imagej.nih.gov/ij/).

## Conflict of interest

The authors declare that they have no competing interests.

## Authors’ contributions

SB and YTeng conceived and planned the study; RT performed most of the experiments; JN collected the samples and extracted total RNAs for qPCR; YTang and YM produced the pear calli from pear fruit; YL and ZY helped with the qPCR assays and the transformation of *Arabidopsis* and pear calli; QY and XY were involved in the bioinformatics analysis; ZW managed the pear trees and collected the fruits from the orchard; and SB, RT and YTeng wrote the manuscript. All authors read and approved the final manuscript.

## Supporting information


**Figure S1** Alignment and phylogenetic tree of PpBBX16.b Phylogenetic tree analysis of PpBBX16 and PpBBX16‐2.
**Figure S2** The phylogenetic analysis of BBX proteins according to their protein sequences.
**Figure S3** Structures of the PpBBX proteins.
**Figure S4** qRT‐PCR analysis of the expression patters of members of BBX gene family.
**Figure S5** Collinearity analysis detected the genome‐wide collinear gene pairs but no BBX genes were detected.
**Table S1** Primer list used in the present work.
