# Supplementary material for: BBX16, a B‐box protein, positively regulates light‐induced anthocyanin accumulation by activating MYB10 in red pear
Source: Plant Biotechnol J. 2019 Apr 14;17(10):1985–97. doi: 10.1111/pbi.13114 (PMC6737026; doi:10.1111/pbi.13114)
Supplement: Supplementary file 1 — Figure S1 Alignment and phylogenetic tree of PpBBX16.b Phylogenetic tree analysis of PpBBX16 and PpBBX16‐2. Figure S2 The phylogenetic analysis of BBX proteins according to their protein sequences. Figure S3 Structures of the PpBBX proteins. Figure S4 qRT‐PCR analysis of the expression patters of members of BBX gene family. Figure S5 Collinearity analysis detected the genome‐wide collinear gene pairs but no BBX genes were detected. Table S1 Primer list used in the present work. [file PBI-17-1985-s001.pdf]

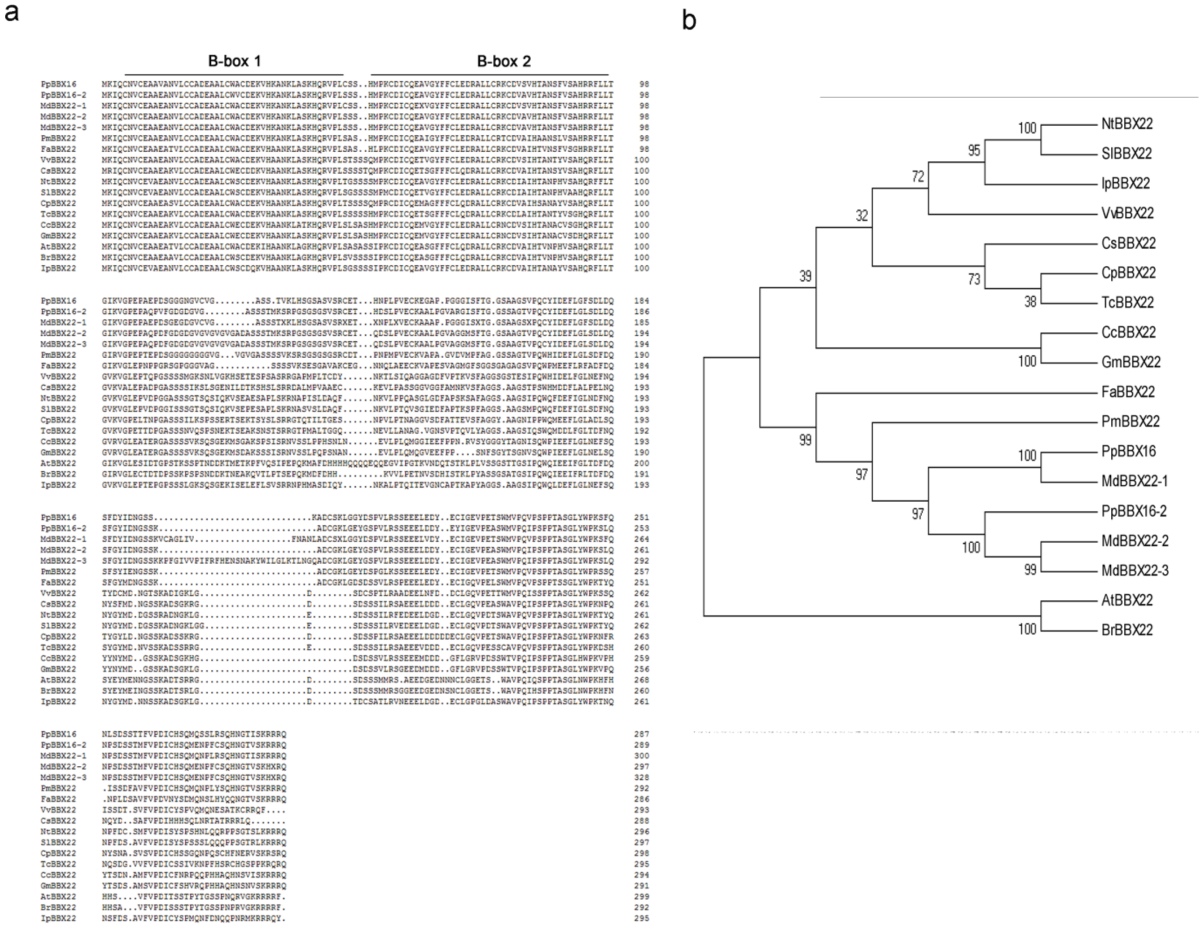

Fig S1. Alignment and phylogenetic tree of PpBBX16. A. Sequence alignment of PpBBX16 with the BBX homologs from other species. Two B-box domains were marked with shade. B. phylogenetic tree analysis of PpBBX16 and the BBX homologs from other species. Pp: *Pyrus pyrifolia*; Md: *Malus domestica* (MdBBX22-1: MDP0000697407; MdBBX22-2: MDP0000298804; MdBBX22-3: MDP0000222881); Pm: *Prunus mume* (XP_008226337.1); Fa: *Fragaria × ananassa* (XP_004294165.1); Vv: *Vitis vinifera* (XP_002283666.1); Cs: *Citrus sinensis* (XP_006477825.1); Nt: *Nicotiana tabacum* (XP_016460367.1); Sl: *Solanum lycopersicum* (XP_004244294.1); Cp:*Carica papaya* (XP_021895033.1); Tc:*Theobroma cacao* (XP_017980396.1); Cc:*Cajanus cajan* (XP_020214951.1); Gm: *Glycine max* (XP_003533890.1); At: *Arabidopsis thaliana* (AEE36125.1); Br: *Brassica rapa* (XP_009106574.1); Ip:*Ipomoea nil* (XP_019194560.1).

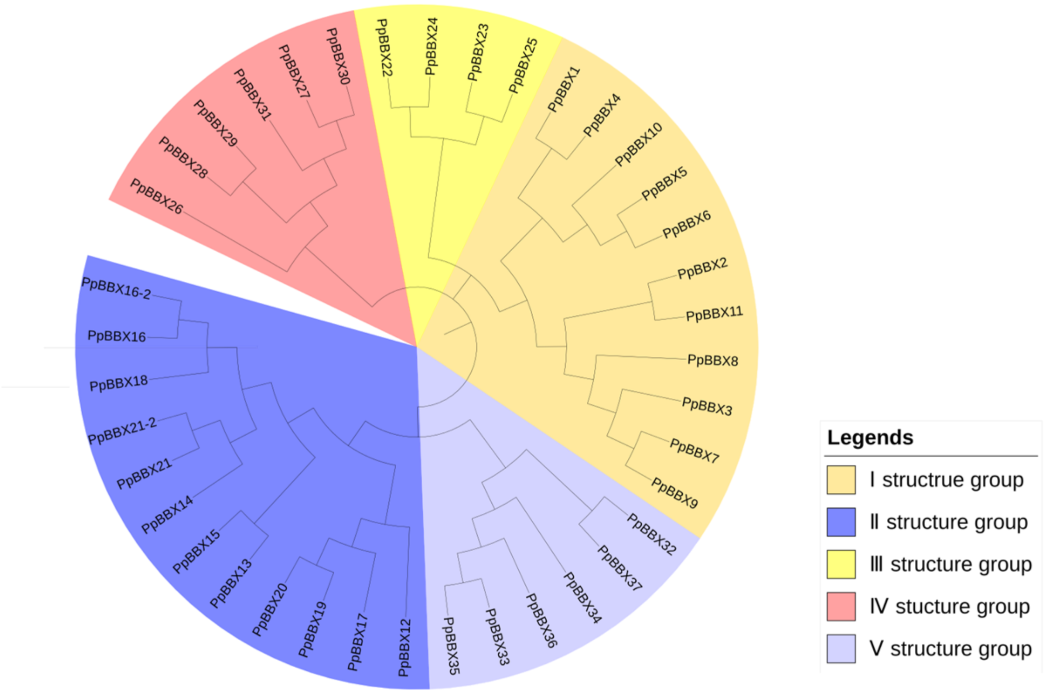

Fig. S2. The phylogenetic analysis of BBX proteins according to their protein sequences.

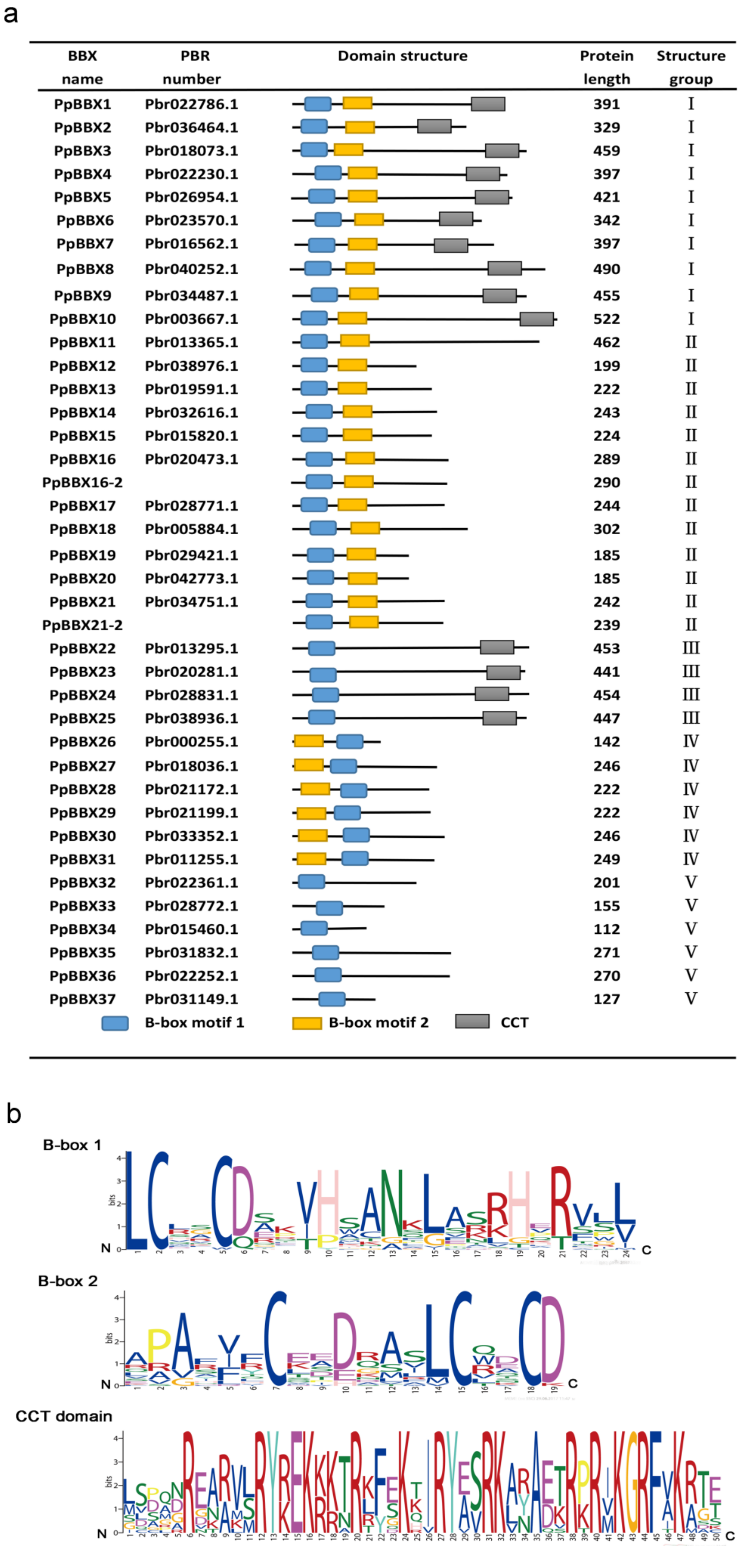

Fig. S3. Structures of the PpBBX proteins. (a) The structures of 39 PpBBX proteins along with protein features, including protein names, the gene id in the published genome and the length of the proteins. (b) The conserved amino acid in B-box 1, B-box 2 and CCT box analyzed by MEME.

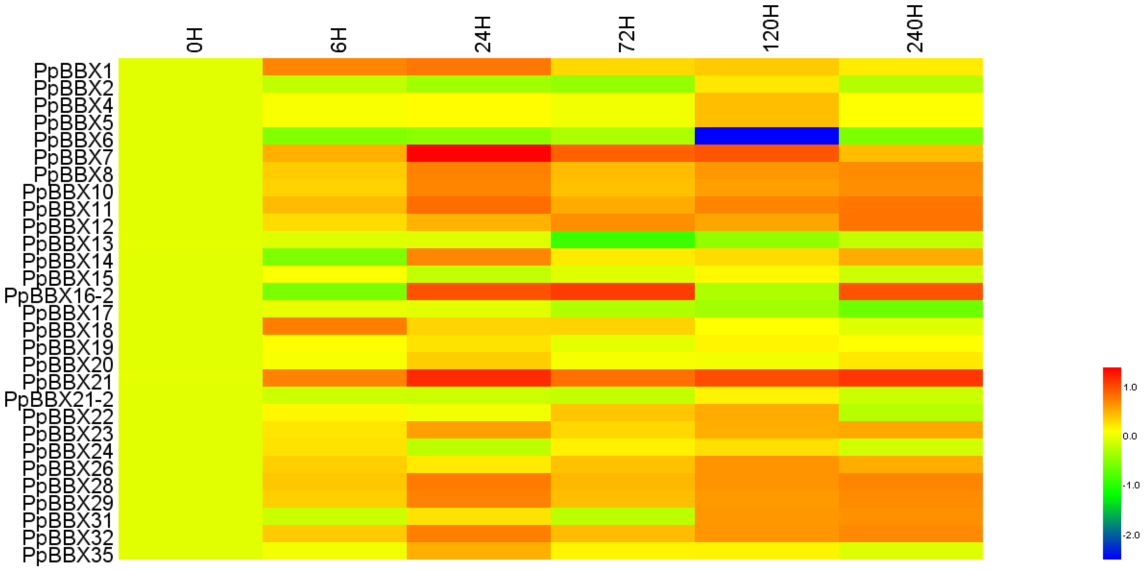

Fig. S4. qRT-PCR analysis of the expression patters of members of BBX gene family. The heatmap showed the log2 relative expression values normalized to 0h. The values are the average of three biological replicates.

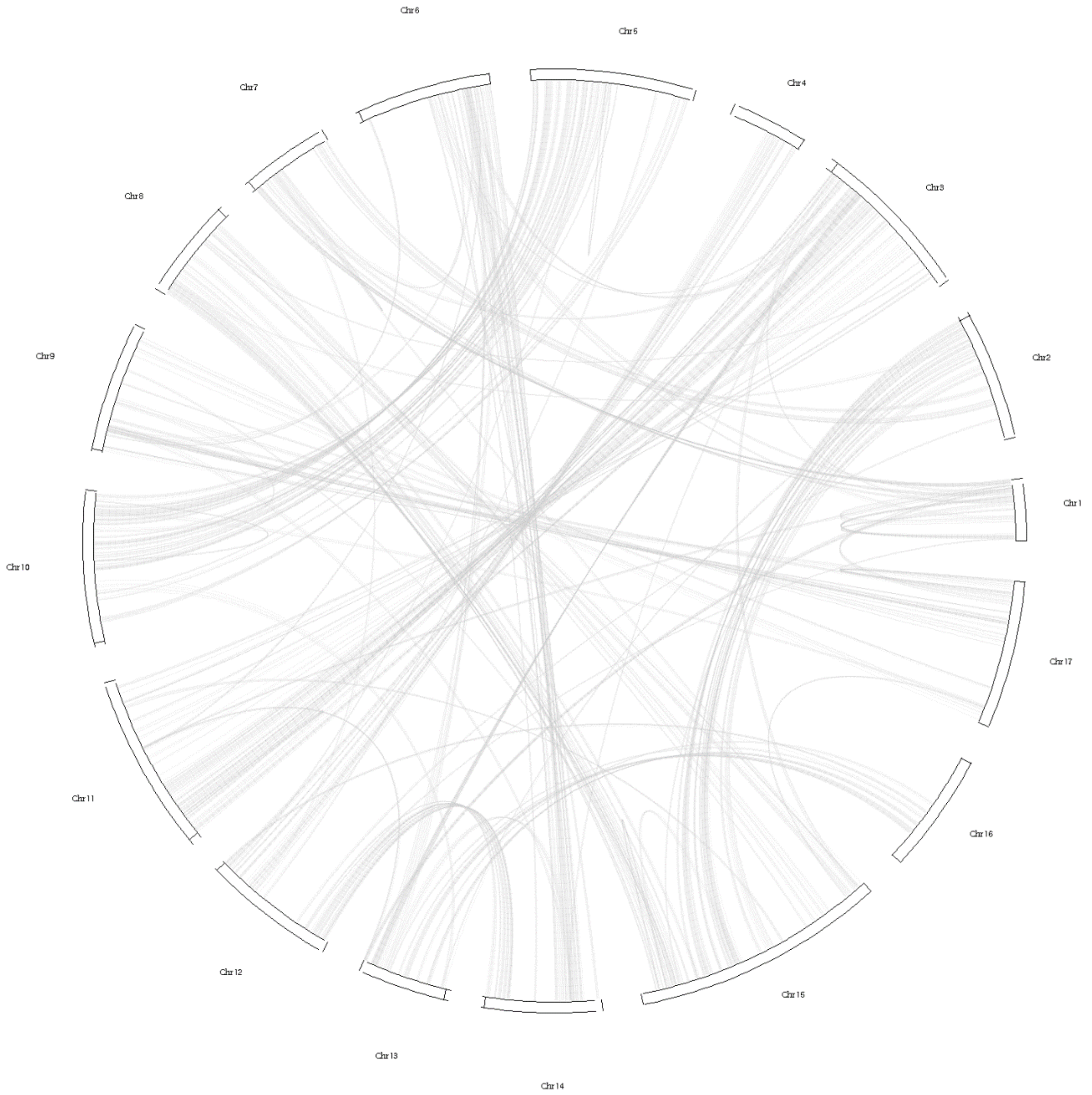

Fig. S5. Collinearity analysis detected the genome-wide collinear gene pairs but no BBX genes were detected.

Table S1. The primers used in the present work.

Primer Names	Forward(5'-3')	Reverse(5'-3')	Usage
PpBBX16	ATGAAGATACAGTGCAACGT	CTAGAATTGCCTACGACGTT	Full-length CDS cloning
PpBBX16-p1301	AGAACACGGGGGACTCTTGACATGAAGATACAGTGCAACGT	GGGGAAATTCGAGCTGGTCACCTAGAATTGCCTACGACGTT	Vector construction
PpBBX16-VIGS	ATTCTGTGAGTAAGGTTACCGCATAACCCGCTGCCGGTGGAG	GCCCGGGCCTCGAGACGCGTGGAAGAATCGGACAGATTCTG	Vector construction
Q-BBX1	CCGAACCAAATACGCAACCA	GACTTCCAGTGCCATCGTTG	qRT-PCR
Q-BBX2	CGTCAACTTCCTCGACAACC	ACATGCCCGTAGTCCAGATC	qRT-PCR
Q-BBX4	CATGAGCGTGTGTGGGTATG	TCCTGCCATGTTCAATTGCC	qRT-PCR
Q-BBX5	AATGGTTGGCAGGAGGAGAA	GAGTCGAAAAGGGCGTTTGT	qRT-PCR
Q-BBX6	GCTATCCGACTCAGTCCCTC	AACGTTGTTGCTGGCTTTCT	qRT-PCR
Q-BBX7	AGAAGGAGGGAAAGCTCACC	CGCTCATTGTTGAATCCGGT	qRT-PCR
Q-BBX8	TGTATCACAGGGCCTTGGTT	TCGGAGCAGGAGGACAAAAT	qRT-PCR
Q-BBX9	TGGATCAGCTGTAGTGGCAA	CGTAAGGCATGCAAATCGGA	qRT-PCR
Q-BBX10	AGTAGGAACAAAGGCGGACA	GACCCTTGACTCGCTTCCTA	qRT-PCR
Q-BBX11	AGGACTTTGCAGCATTGTGG	TCCGGGTGAGAGAATTTGCT	qRT-PCR
Q-BBX12	AGGCAGAGAGTTGAGTTCCC	TTGCTCGCGTATTCTTTGGG	qRT-PCR
Q-BBX13	TGTCAGGAAAGGCGAGGATT	GAGCAGGCTGATGAAGTTGG	qRT-PCR
Q-BBX14	TGGGTGTTTTCGGCAAAGAG	GCCCTCCAACAATTCGATCC	qRT-PCR
Q-BBX16	ACGTTTGTTCCAGACATTTGCCA	CAGGAGATGATGGGAAGTGGTAAGA	qRT-PCR
Q-BBX16-2	CCACATGCCCAAATGCGATA	GACCCAGGACGTGACTTCAT	qRT-PCR
Q-BBX17	GCAAGATCTCGGGGTTTGTG	TTCATGAATTGCGGCTCTGC	qRT-PCR
Q-BBX18	AATGCCCCGTTTGGTTTCTC	CCACATTCTGTTGCTGGCTT	qRT-PCR
Q-BBX21	GGTGTTGATGACTTGCTGCA	CATCGTCCTCCAGCATTTCG	qRT-PCR
Q-BBX21-2	CCTTCAGAGCCTCTCCAACA	CCTTGGTACTACTGGAGGCC	qRT-PCR
Q-BBX22	GGCGGACGAAGATGCAATAG	TGTACCCCGATTCCGTTCAA	qRT-PCR
Q-PpBBX23	GCACATGGAGATTCAGCAGG	ATCGTGTCCTTCGCTTCTCT	qRT-PCR
Q-BBX26	AACTCTGCTCTGCCATGTCT	CTTCATCTTCTTCCGCACCG	qRT-PCR
Q-BBX28	ATGGTGAGGAGGAGGAGGAT	GAACCGAACGCCTTCATCTC	qRT-PCR
Q-BBX29	ATGGTGAGGAGGAGGAGGAT	GAACCGAACGCCTTCATCTC	qRT-PCR
Q-BBX31	AGGGCGGAGATAATCAGGTG	GGAGATCGTCGACCTGAGAG	qRT-PCR
Q-BBX32	GTCTCTCCAACTGCAGTCCT	GAACAGGAACGACACTTGCA	qRT-PCR
Q-BBX35	AGAAGCTTGGGGTGAATGGT	CTCCTCCACCCGTCTCAAAT	qRT-PCR
Q-BBX36	TGGTTTCAACGGCGTCAAAT	TCGACCCGTCTCAGATTCTG	qRT-PCR
Q-AtCHS	AGCTGATGGACCTGCAGGCATCTTGGC	TGCATGTGACGTTTCCGAATTGTCGAC	qRT-PCR
Q-AtCHI	ATGTCTTCATCCAACGCCTGCGCC	GACGGTGAAGATCACGAATTTACC	qRT-PCR
Q-AtDFR	ATGGTTAGTCAGAAAGAGACCGTGTGTG	CGTTTCGCAAATCAAGAAGATGTTGTAC	qRT-PCR
Q-AtF3H	ATGGCTCCAGGAACTTTGACTGAGCTA	GATCTGACGGCAGATCTCTCCTCTTTT	qRT-PCR
Q-AtLDOX	TGGGTCACTGCAAAATGTGT	CGGAGACTCAACACTCACCA	qRT-PCR
Q-PpPAL	TCTGCCAGGGAAAGATTATCG	TGAAGTTGAATGGAATGGAATGC	qRT-PCR
Q-PpCHS	GGGTGTACTCTTCGGATTTGG	AAAGGCGGAAACAATACATATACG	qRT-PCR
Q-PpCHI	GAACGGGTGCAAGGAATCTA	AACAGGAGTCCCTCCCAAGT	qRT-PCR
Q-PpF3H	GGAGAAAGACAAAGTGGAGATAAAGC	ACAAGAAGTGGAAAGGCAAAGTTAC	qRT-PCR

Q-PpDFR	ACTGAGGCTGCTGAGGAGAG	TCAAATCCAAGCTGGTAAATGT	qRT-PCR
Q-PpANS	AGTTGTTCAGGAAAAGCCAAGAGG	ACAAAGCAGGCAGATAGGAGTAGC	qRT-PCR
Q-PpUFGT	CTGGAACCTGAAGTTGTGAATCTG	AGCCACTCTAAGCAACCACTATC	qRT-PCR
Q-PpMYB10	CAGCAGAAGATTTAAGTACGCCATC	TTCTAACAAGGTCTCCCACCAATC	qRT-PCR
Q-PpbHLH3	ATGGCTCAGAATCATGAGAGGGTG	TCAGCACTTACCAGCAATTTTC	qRT-PCR
PpWD40	CGGTGTATTCGCCTCCGTCTC	GACTGGTGCCTCTGCAACTCG	qRT-PCR
